# Freiwilligkeit bei Anfragen nach Suizidassistenz

**DOI:** 10.1007/s00103-026-04190-1

**Published:** 2026-01-29

**Authors:** Esther Braun, Tanja Henking, Christin Hempeler, Jan Schildmann, Jakov Gather

**Affiliations:** 1 Juniorprofessur für Medizinische Ethik mit Schwerpunkt auf Digitalisierung, Fakultät für Gesundheitswissenschaften Brandenburg, Universität Potsdam, Am Mühlberg 9, 14476 Potsdam, Deutschland; 2 Department of Philosophy, University of Oxford, Oxford, Großbritannien; 3 Institut für Angewandte Sozialwissenschaften (IFAS), Technische Hochschule Würzburg-Schweinfurt, Würzburg, Deutschland; 4 Institut für Medizinische Ethik und Geschichte der Medizin, Ruhr-Universität Bochum, Bochum, Deutschland; 5 Institut für Geschichte und Ethik der Medizin, MLU Halle-Wittenberg, Halle/Saale, Deutschland; 6 Klinik für Psychiatrie, Psychotherapie und Präventivmedizin, LWL-Universitätsklinikum, Ruhr-Universität Bochum, Bochum, Deutschland

**Keywords:** Informierte Einwilligung, Freiverantwortlichkeit, Unzulässige Einflussnahme, Druck, Ethik, Informed consent, Autonomy, Undue influence, Pressure, Ethics

## Abstract

Freiwilligkeit bezeichnet die Freiheit einer Entscheidung von unzulässiger Einflussnahme oder Druck. Sie ist ein Element der Freiverantwortlichkeit von Entscheidungen über assistierten Suizid und damit ein wichtiger Beurteilungsgegenstand im Umgang mit Anfragen nach Suizidassistenz. Der vorliegende Beitrag widmet sich der Frage, welche Faktoren die Freiwilligkeit von solchen Anfragen potenziell einschränken können.

Anhand der medizinethischen Literatur sowie empirischer Studien aus verwandten Kontexten werden u. a. Mitarbeitende im Gesundheitswesen, Sterbehelfer*innen und Angehörige als zentrale Akteure aufgezeigt, die bei Entscheidungen über assistierten Suizid relevant sein können. Mögliche Formen interpersonaler Einflussnahme können Drohungen, Angebote, zwischenmenschliche Beeinflussung und Überredung sein. Zusätzlich stellen strukturelle Rahmenbedingungen – etwa sozioökonomische Notlagen – mögliche Einflussfaktoren auf die Freiwilligkeit dar. Bei der Beurteilung der Freiverantwortlichkeit eines Suizidentschlusses sollte daher systematisch geprüft werden, ob solche Einflussfaktoren vorliegen.

Angesichts der bislang geringen empirischen Datenlage bedarf es dringend weiterer Forschung in diesem Bereich. Dies ist auch für die Entwicklung von Leitlinien zur Beurteilung der Freiwilligkeit bei Anfragen nach assistiertem Suizid essenziell.

## Abstract (en)

Voluntariness refers to the freedom of a decision from undue influence or pressure. It is one component of an autonomous decision for assisted suicide and, therefore, an important factor to consider when assessing requests for assisted suicide. This article examines which factors can potentially impact the voluntariness of such requests.

Based on the medical ethics literature and empirical studies from related contexts, relatives, individuals providing suicide assistance, and healthcare workers are identified, among others, as key actors who may be relevant in decisions about assisted suicide. Possible forms of interpersonal influence include threats, offers, interpersonal leverage, and persuasion. In addition, structural constraints—such as socioeconomic hardship—can potentially influence voluntariness. When assessing whether requests for assisted suicide are autonomous, the potential presence of such factors should be systematically examined.

Given the limited availability of empirical data to date, there is an urgent need for further research in this area. This is also essential for the development of guidelines for assessing the voluntariness of requests for assisted suicide.

## Einleitung

Anfragen nach Suizidassistenz stellen für im Gesundheitswesen tätige Personen eine Herausforderung dar – auch, da etablierte Standards für den Umgang mit solchen Anfragen noch weitgehend fehlen. An der grundsätzlichen rechtlichen Zulässigkeit von Suizidassistenz unter bestimmten Bedingungen besteht hingegen seit der Entscheidung des Bundesverfassungsgerichts (BVerfG) aus dem Jahr 2020 kein Zweifel mehr [1]. Dabei ist aus rechtlicher Sicht zentral, dass die Entscheidung zum Suizid freiverantwortlich getroffen wird [2, 3].^1^

Die Entscheidung des BVerfG gibt bereits wertvolle Hinweise dazu, wie die Freiverantwortlichkeit im konkreten Fall beurteilt werden kann. Hierbei greift sie auf die in der rechtswissenschaftlichen Literatur vertretene Einwilligungslösung zurück [6–8], der sich im Jahr 2019 bereits der Bundesgerichtshof angeschlossen hatte [9]. Es kommt dabei darauf an, dass die suizidwillige Person die Tragweite ihres Entschlusses erkennen (Einsichtsfähigkeit) und beurteilen (Urteilsfähigkeit) kann. Das BVerfG hebt außerdem die Kriterien der „gewissen Dauerhaftigkeit“ und „inneren Festigkeit“ des Suizidentschlusses hervor. Zudem gehe der Suizidentschluss dann auf einen autonom gebildeten, freien Willen zurück, wenn die Person ihre Entscheidung auf der Grundlage einer realitätsbezogenen, am eigenen Selbstbild ausgerichteten Abwägung des Für und Wider in Kenntnis der entscheidungsrelevanten Tatsachen trifft. Dabei verweist das BVerfG auf die Vergleichbarkeit mit der Einwilligung in eine Heilbehandlung. Die Willensbildung muss ferner mangelfrei sein – sie darf also beispielsweise nicht auf falschen Annahmen oder Täuschung beruhen. Schließlich dürfe der Willensentschluss keiner unzulässigen Einflussnahme oder Druck unterliegen.^2^

In diesem Beitrag wird die Bedeutung solcher Formen von unzulässiger Einflussnahme und Druck für die Freiverantwortlichkeit einer Entscheidung betrachtet. Diese Aspekte werden in der Medizinethik unter dem Begriff der *Freiwilligkeit* diskutiert. Freiwilligkeit ist neben Information und Einwilligungsfähigkeit ein zentrales Element einer informierten Einwilligung, die eine wesentliche normative Voraussetzung für die Zulässigkeit einer medizinischen Maßnahme darstellt [11, 12]. In der Medizinethik wird Freiwilligkeit unter anderem bei Fragestellungen diskutiert, bei denen es (auch) um finanzielle Kompensationen geht, z. B. bei Forschungsteilnahme, Leihmutterschaft oder Organspende [13–15], sowie im Rahmen von Einflussnahme innerhalb persönlicher oder therapeutischer Beziehungen, etwa im psychiatrischen Behandlungskontext [16–18]. In Bezug auf die Freiwilligkeit von Entscheidungen über assistierten Suizid existieren bisher noch keine umfassenden empirischen oder ethischen Untersuchungen, weshalb im Folgenden auf Forschungsergebnisse aus den genannten Bereichen Bezug genommen wird.

Ziel dieses Beitrags ist es, aus einer interdisziplinären Perspektive einen ersten Überblick über mögliche Einschränkungen der Freiwilligkeit im Kontext der Suizidassistenz zu geben. Hierbei soll nicht abschließend geklärt werden, ab wann genau eine Entscheidung zum assistierten Suizid als *unfreiwillig* angesehen werden sollte. Auch soll nicht suggeriert werden, dass eine Suizidassistenz per se ethisch gerechtfertigt ist, wenn keine Einschränkung der Freiwilligkeit vorliegt. Stattdessen soll für verschiedene Formen der äußeren Einflussnahme sensibilisiert werden, die Entscheidungen für einen assistierten Suizid möglicherweise unfreiwillig machen können, um diese zu erkennen und möglichst zu vermeiden.

Im Folgenden werden zunächst *Akteure *aufgezeigt, die im Kontext des assistierten Suizids eine wichtige Rolle spielen und Einfluss auf Entscheidungen nehmen können. Im Anschluss werden mögliche *Formen* der interpersonalen Einflussnahme beschrieben (Drohungen, Angebote, zwischenmenschliche Beeinflussung und Überredung). Zudem wird auf den möglichen Einfluss *struktureller Faktoren* eingegangen. Abschließend wird erörtert, worauf bei der Beurteilung von Freiwilligkeit im Kontext von Anfragen nach Suizidassistenz geachtet werden sollte.

## Mögliche Einschränkungen der Freiwilligkeit

Menschen unterliegen in allen Entscheidungen äußeren Einflüssen. Dazu gehören sowohl Einflüsse durch andere Personen als auch strukturelle Einflüsse, wie beispielsweise gesellschaftliche Erwartungen [19]. Allein die Tatsache, dass solche äußeren Faktoren bei einer Entscheidung eine Rolle spielen, ist noch kein Hinweis auf eine fehlende Freiwilligkeit [20]. Zu klären ist vielmehr, wann durch eine äußere Einflussnahme *unzulässiger *Druck auf eine Person ausgeübt und damit die Freiwilligkeit ihrer Entscheidung beeinträchtigt wird.

Ähnlich wie auch die Einsichts- und Urteilsfähigkeit stellt Freiwilligkeit ein graduelles Konzept dar – das heißt, eine Person kann bei einer Entscheidung *mehr oder weniger* äußeren Einflüssen oder Druck ausgesetzt und die Freiwilligkeit einer Entscheidung kann dadurch *mehr oder weniger *beeinträchtigt sein. Am Ende bedarf es aber eines kategorialen Urteils: Es muss geklärt werden, ob eine Entscheidung einer Person hinreichend freiwillig ist oder nicht. Ab wann genau eine Einflussnahme eine Entscheidung *unfreiwillig *und damit *nicht *selbstbestimmt (bzw. nicht freiverantwortlich) macht, wird in Medizinethik und Philosophie kontrovers diskutiert [14].

Es besteht breiter Konsens darüber, dass Entscheidungen, die durch *Zwang* herbeigeführt wurden, nicht freiwillig sind [11, 21]. Zwang liegt vor, wenn eine Person Kontrolle über die Handlungen oder den Willen einer anderen Person ausübt und somit ihre Handlungsoptionen bestimmt. In der Literatur finden sich jedoch unterschiedliche Positionen dazu, welche Formen der kommunikativen Einflussnahme als Zwang angesehen werden sollten. Gemäß einer einflussreichen philosophischen Position kann ausschließlich durch Drohungen Zwang ausgeübt werden [13, 22]. Andere Autor*innen argumentieren jedoch, dass nicht nur durch Drohungen, sondern auch durch Angebote oder andere Formen der Einflussnahme Zwang ausgeübt werden kann [16, 23, 24].

Stellenweise wird in der Medizinethik angenommen, dass auch „interne“ psychologische Faktoren die Freiwilligkeit einschränken können, etwa im Rahmen psychischer Erkrankungen [11]. Viele „interne“ psychologische Faktoren sind allerdings auch über das Kriterium der Einsichts- und Urteilsfähigkeit abgedeckt. Wenn die Entscheidung einer Person zum Suizid zum Beispiel durch objektiv nicht angemessene Hoffnungslosigkeit in Bezug auf die Wirksamkeit sämtlicher Behandlungsmöglichkeiten beeinflusst ist, könnte dies die Einsichtsfähigkeit einschränken [25].

Weitere mögliche Beeinträchtigungen der Freiwilligkeit durch „interne“ Faktoren, etwa bei Sucht- oder Zwangserkrankungen, sind im Kontext des assistierten Suizids weniger relevant. Im Folgenden wird daher die Diskussion *externer *Faktoren in den Mittelpunkt gestellt, die die Freiwilligkeit von Entscheidungen beeinflussen können, vorrangig mögliche äußere Einflussnahmen durch andere Personen.

### Relevante Akteure im Kontext der Suizidassistenz

#### Mitarbeitende im Gesundheitswesen

Mitarbeitende im Gesundheitswesen wie Ärzt*innen oder Pflegende, aber auch Fachpersonal aus anderen therapeutischen Berufen nehmen häufig eine besondere Rolle bei Entscheidungen über Suizidassistenz ein. Sie sind einerseits in die medizinische Versorgung von Personen eingebunden, die im Verlauf ihrer Erkrankungen einen assistierten Suizid erwägen. Andererseits sind Mitarbeitende im Gesundheitswesen oftmals diejenigen, die für eine Suizidassistenz angefragt werden und diese gegebenenfalls auch leisten [26].

Empirische Untersuchungen aus Deutschland weisen darauf hin, dass Ärzt*innen aus unterschiedlichen Fachgebieten mit Anfragen nach assistiertem Suizid konfrontiert sind [27–30]. In daraus folgenden Gesprächen über assistierten Suizid können Ärzt*innen und andere Mitarbeitende im Gesundheitswesen informieren, beraten und mögliche Alternativen aufzeigen [31].

#### Sterbehelfer*innen

Empirische Daten deuten darauf hin, dass Suizidassistenz in Deutschland aktuell primär von sogenannten Sterbehilfeorganisationen geleistet wird [32]. Die Organisationen verweisen ihre Mitglieder an Sterbehelfer*innen, wobei es sich laut Informationen der Sterbehilfeorganisation „Deutsche Gesellschaft für Humanes Sterben“ beispielsweise um Ärzt*innen und Jurist*innen handelt [33]. Diesen kommt einerseits eine wichtige Rolle bei der Beurteilung der Freiwilligkeit von Suizidwünschen zu. Andererseits können sie in Beratungsgesprächen Informationen zu Suizidassistenz sowie mögliche Handlungsalternativen vermitteln. Dabei ist zu beachten, dass es bei den beteiligten Akteuren teils Überlappungen gibt: So können Ärzt*innen sowohl im Rahmen solcher Vereine als auch unabhängig davon Suizidassistenz leisten.

#### Angehörige

Internationale Forschungsergebnisse weisen darauf hin, dass Angehörige bei Entscheidungen über assistierten Suizid häufig eine wichtige Rolle spielen [34]. Dies kann sich einerseits auf Familienangehörige beziehen, aber auch auf andere den Betroffenen nahestehende Personen, wie z. B. Freund*innen. Angehörige werden häufig in den Entscheidungsprozess einbezogen [35]. Zudem kann die Sorge, Angehörigen finanziell oder in Bezug auf die Pflege zur Last zu fallen, eine relevante Abwägung bei Entscheidungen über assistierten Suizid sein [34, 36, 37]. Eine besondere Konstellation besteht in Fällen, in denen beispielsweise Ehepartner*innen gemeinsam Suizidassistenz anfragen [38].

Internationale Forschungsergebnisse zeigen, dass auch Ärzt*innen die Einstellungen von Angehörigen berücksichtigen, um zu entscheiden, ob sie Suizidhilfe leisten [39, 40], beispielsweise indem sie mögliche Auswirkungen eines assistierten Suizids auf Angehörige in ihre Entscheidungen einbeziehen. Diese Aspekte können auch in Aufklärungs- und Informationsgesprächen thematisiert werden.

#### Einfluss der Akteure auf die Freiwilligkeit von Entscheidungen

Dass bei einer Entscheidung über einen assistierten Suizid andere Personen einbezogen werden, ist für sich genommen kein Hinweis darauf, dass die Entscheidung nicht freiwillig getroffen wird. Für die Freiwilligkeit einer Entscheidung ist vielmehr relevant, ob andere Personen diese auf eine ethisch problematische Weise beeinflussen, indem sie bewusst oder unbewusst Druck ausüben. Die genannten Akteure sollten sich daher bewusst sein, dass sie bei Informations- und Beratungsgesprächen über assistierten Suizid sowie bei informellen Gesprächen – auch unabsichtlich – Druck ausüben können.

### Formen interpersonaler Einflussnahme

In der Medizinethik werden unterschiedliche Formen der Einflussnahme diskutiert, durch die Druck auf Personen ausgeübt werden kann, um sie zu einer bestimmten Entscheidung zu bewegen. Im Folgenden wird ein Überblick über unterschiedliche Formen der interpersonalen Einflussnahme gegeben, die – auch in Kombination miteinander – im Kontext des assistierten Suizids potenziell eine Rolle spielen könnten: Drohungen, Angebote, zwischenmenschliche Beeinflussung und Überredung. Diese Klassifikation folgt einem etablierten Modell von Behandlungsdruck in der Psychiatrie [16, 17].

Bei allen diskutierten Formen der Einflussnahme ist einerseits denkbar, dass sie angewendet werden, um eine Person zum assistierten Suizid zu bewegen, aber auch, um sie von einer Entscheidung für einen assistierten Suizid abzubringen. In diesem Beitrag wird die Beeinflussung zum assistierten Suizid hin betrachtet.

Weiterhin ist bei allen aufgeführten Formen der Einflussnahme einerseits denkbar, dass diese absichtlich und bewusst mit der Intention angewendet werden, die Person zu einer Entscheidung zu bewegen. Andererseits ist jedoch auch vorstellbar, dass durch Aussagen oder Verhalten *unabsichtlich *oder *unbewusst* Druck auf eine Person ausgeübt wird [16]. Hierbei ist relevant, wie eine Interaktion subjektiv wahrgenommen wird, also ob die Person gefühlt unter Druck steht [41, 42].

Da bisher kaum empirische Literatur zu Freiwilligkeit in Bezug auf assistierten Suizid existiert, bezieht sich die nachfolgende Diskussion auf hypothetische Beispiele. Orientierung bietet hierbei die empirische Forschung aus anderen Bereichen, in denen die Anwendung verschiedener Formen der Einflussnahme bereits untersucht wurde – insbesondere im Kontext von Behandlungsdruck in der Psychiatrie [16, 18, 43], aber auch in der Geburtshilfe [44], im Rahmen von Lebendorganspenden [45, 46] sowie bei Gesprächen über lebenserhaltende Maßnahmen [47, 48]. Ob diese Formen der Einflussnahme beim assistierten Suizid tatsächlich eine Rolle spielen, muss durch empirische Forschung untersucht werden – die nachfolgende Übersicht soll lediglich einen Eindruck davon vermitteln, wie solche Formen aussehen könnten, und für diese sensibilisieren.

#### Drohungen

Eine starke Form der Einflussnahme stellen *Drohungen *dar. In der Regel wird angenommen, dass eine Entscheidung nicht freiwillig, sondern erzwungen ist, wenn sie maßgeblich von einer direkten Drohung beeinflusst wird, der die Person nicht standhalten kann. Gemäß den einflussreichen Definitionen von Nozick [22] und Wertheimer [13] liegt dann eine Drohung vor, wenn eine Person einer anderen ankündigt, sie schlechter zu stellen als moralisch angemessen wäre, um sie zu einer bestimmten Entscheidung zu bewegen.

In Bezug auf Suizidassistenz sind Drohungen einerseits denkbar, um eine Person davon abzuhalten, ihren Wunsch weiterhin zu äußern. Beispielsweise könnte ein Arzt androhen, eine Patientin nicht mehr zu behandeln, wenn sie weiterhin einen Wunsch nach assistiertem Suizid äußert. Hierbei handelt es sich um eine Drohung, da die angekündigten Konsequenzen die Patientin schlechter stellen als moralisch erwartbar wäre – denn die Patientin hat ein Recht darauf, medizinisch versorgt zu werden, auch wenn ihr Wunsch nach einem assistierten Suizid der Haltung des Behandlers widerspricht.

Andererseits ist auch vorstellbar, eine Person durch eine Drohung zum assistierten Suizid bewegen zu wollen. Beispielsweise wäre denkbar, dass Mitarbeitende im Gesundheitswesen androhen, die Pflege einer Person einzustellen, wenn sie sich nicht für einen assistierten Suizid entscheidet. Hierbei handelt es sich jedoch um ein drastisches – und gegebenenfalls strafbares – Beispiel.

Weiterhin sind auch subtilere Drohungen vorstellbar – beispielsweise könnte eine Person im Kontext eines Gesprächs über assistierten Suizid ankündigen, dass sie die Kosten für die Pflege ihrer Tante in Zukunft nicht mehr übernehmen könne. Aus philosophischer Sicht handelt es sich hier eher nicht um eine „echte“ Drohung, zumindest wenn nicht davon ausgegangen wird, dass die Betroffene ein moralisches Recht darauf hat, dass ihre Nichte die Kosten für ihre Pflege übernimmt. Jedoch ist vorstellbar, dass auch solche Äußerungen Entscheidungen zum assistierten Suizid deutlich beeinflussen können.

#### Angebote

Ein Angebot ist dadurch gekennzeichnet, dass Personen durch dessen Annahme *besser*gestellt werden. Im Gegensatz zu einer Drohung kann ein Angebot abgelehnt werden, ohne dass hieraus negative Konsequenzen für die Person entstehen. Dennoch wird diskutiert, ob auch Angebote unter gewissen Umständen Zwang ausüben können [23, 24]. Im Kontext der Suizidassistenz erscheint besonders relevant, ob durch das Anbieten der Option des assistierten Suizids unzulässiger Druck ausgeübt werden kann.

Manche rechtlichen Regelungen zur Suizidassistenz verbieten es, das Thema Suizidassistenz bei Patient*innen proaktiv anzusprechen, die nicht ausdrücklich danach gefragt haben, etwa im australischen Staat Victoria. Hierdurch sollen eine unzulässige Einflussnahme und Einschränkung der Freiwilligkeit verhindert werden [49].

In anderen Regelungen hingegen werden Suizidassistenz oder Tötung auf Verlangen als „Behandlungsoption“ und Teil der medizinischen Versorgung verstanden, über die Patient*innen informiert werden können oder sogar sollten. So schreibt eine kanadische Richtlinie Ärzt*innen eine professionelle Verpflichtung zu, die Thematik bei Patient*innen, für die Suizidassistenz eine rechtlich zulässige Möglichkeit wäre, proaktiv anzusprechen [50].

Es wird jedoch diskutiert, dass solche „Hinweise“ bei der Behandlung von Patient*innen auch als implizite Aufforderung verstanden werden können, die Druck ausüben und damit potenziell die Freiwilligkeit einschränken können [51]. Manche Autor*innen argumentieren, dass Hinweise auf die Möglichkeit von assistiertem Suizid zu Hoffnungslosigkeit bei Patient*innen führen könnten, wenn daraus zum Beispiel geschlossen wird, es bestünden keine weiteren Behandlungsoptionen für ihre Erkrankung [52].

Angebote oder Hinweise auf assistierten Suizid könnten zudem in unterschiedlichen Situationen, die über den unmittelbaren Behandlungskontext hinausgehen, eine Rolle spielen. In Kanada wurden beispielsweise Fälle kritisiert, in denen Personen auf die Möglichkeit von Suizidassistenz bzw. Tötung auf Verlangen hingewiesen wurden, als sie versuchten, bei den zuständigen Behörden Hilfsmittel wie etwa eine Rollstuhlrampe zu beantragen [52]. Vorstellbar wären auch Hinweise auf die Möglichkeit von assistiertem Suizid in Pflegeheimen. Hierdurch könnten sich betroffene Personen zur Suizidassistenz gedrängt fühlen, was die Freiwilligkeit ihrer Entscheidung beeinträchtigen könnte.

#### Zwischenmenschliche Beeinflussung

Eine weitere denkbare Form der interpersonalen Einflussnahme besteht in sogenannter zwischenmenschlicher Beeinflussung. Diese umfasst die Ankündigung von Veränderungen in emotionalen Einstellungen [16, 17]. So könnten Angehörige etwa deutlich machen, dass sie eine Entscheidung für einen assistierten Suizid befürworten würden und Veränderungen in der Beziehung oder emotionale Konsequenzen in Aussicht stellen, wenn die Person sich nicht für eine Suizidassistenz entscheidet. Dies könnte beispielsweise die Ankündigung umfassen, dass die Angehörigen wütend, enttäuscht oder emotional überfordert wären, wenn die betroffene Person sich nicht für eine Suizidassistenz entscheidet und sie diese weiter „leiden“ sehen müssten. Neben Angehörigen könnten auch Mitarbeitende im Gesundheitswesen, die in enger Beziehung zu anfragenden Personen stehen, zwischenmenschliche Beeinflussung ausüben.

#### Überredung

Eine Einflussnahme wird als Überredung bezeichnet, wenn eine Person versucht, eine andere Person mit (scheinbar) rationalen Argumenten zu einer bestimmten Handlungsoption zu bewegen [17]. Es ist vorstellbar, dass sich eine Person zum Suizid gedrängt fühlt, wenn ihr Argumente für die Inanspruchnahme einer Suizidassistenz präsentiert werden: beispielsweise wenn einer Person erklärt wird, dass durch einen assistierten Suizid die finanzielle Belastung der Angehörigen durch ihre Pflege deutlich reduziert würde oder dass viele Personen in einer ähnlichen Lage assistierten Suizid als eine gute Option ansehen.

Forschung zur Kommunikation von Ärzt*innen über Behandlungsoptionen am Lebensende zeigt, dass die Art und der Kontext, in dem Argumente für und gegen unterschiedliche Optionen präsentiert werden, die Entscheidungen von Patient*innen deutlich beeinflussen können [47, 53]. Dies kann beispielsweise durch eine unausgewogene Darstellung der Vor- und Nachteile bestimmter Handlungsoptionen erfolgen oder durch Verweise darauf, wie sich andere Patient*innen in ähnlichen Situationen typischerweise entscheiden [48, 53]. Solche Verweise können bestimmte Handlungsoptionen erwünschter erscheinen lassen, was Patient*innen unter Umständen dazu drängen kann, eine bestimmte Entscheidung zu treffen [48]. Hierbei ist auch zu berücksichtigen, dass Patient*innen (auch impliziten) Empfehlungen von Ärzt*innen häufig besonderes Gewicht geben und durch diese stark beeinflusst werden können [47].

### Strukturelle Faktoren

Auf struktureller Ebene wird schon lange diskutiert, dass infolge der Legalisierung von assistiertem Suizid eine gesellschaftliche Erwartungshaltung gegenüber bestimmten Personengruppen – zum Beispiel pflegebedürftigen Menschen – entstehen könnte, assistierten Suizid in Anspruch zu nehmen [54, 55]. Wenn betroffene Personen annehmen, es sei gesellschaftlich erwartet, Suizidassistenz zu nutzen, könnte sich dies einerseits darauf auswirken, wie die oben beschriebenen interpersonalen Einflüsse wahrgenommen werden, und diese Einflüsse verstärken [16]. Andererseits wird auch diskutiert, ob strukturelle Faktoren selbst die Freiwilligkeit einschränken können, ohne dass eine andere Person explizit Einfluss nimmt.

Hierbei wird vor allem der Einfluss gesellschaftlich verursachter Notlagen diskutiert, beispielsweise durch fehlende palliative Versorgungsmöglichkeiten [56] oder fehlende pflegerische oder finanzielle Unterstützung von Personen mit Behinderungen und chronischen Erkrankungen [55, 57, 58]. Lange wurde größtenteils angenommen, dass sozioökonomische Notlagen keine Rolle für Entscheidungen zum assistierten Suizid spielen, da empirische Daten aus verschiedenen Ländern dafür sprechen, dass assistierter Suizid überwiegend von Personen mit hohem Bildungsstand und sozioökonomischen Status angefragt wird [59, 60]. Durch aktuelle Entwicklungen in verschiedenen Ländern erfährt die Rolle von strukturellen Faktoren bei Entscheidungen für assistierten Suizid jedoch zunehmend Aufmerksamkeit.

Laut verschiedener medialer Berichte ist es beispielsweise in Kanada in den letzten Jahren mehrfach zu Fällen gekommen, in denen Personen mit chronischen Erkrankungen oder Behinderungen aufgrund sozioökonomischer Notlagen eine Tötung auf Verlangen anfragten und zum Teil auch erhielten [61–63]. In den entsprechenden Fällen bestand der Wunsch nach Tötung auf Verlangen beispielsweise aufgrund von Armut, inadäquater Wohnsituation oder drohender Wohnungslosigkeit. Laut Zeitungsberichten war die unzureichende Unterstützung – und nicht die medizinische Diagnose – der hauptsächliche Grund für diese Anfragen [62, 63]. Einige Autor*innen sehen diese Fälle zwar als tragisch, aber als autonome Entscheidungen an, weshalb die entsprechenden Wünsche respektiert werden sollten [57]. Andere wiederum verweisen darauf, dass teils auch ungerechte strukturelle Gegebenheiten Zwang vermitteln und Entscheidungen unfreiwillig machen können [52].

In Deutschland weist die bisher eingeschränkte empirische Datenlage darauf hin, dass die Mehrzahl der Suizidanfragen von Personen mit einem hohen sozioökonomischen Status stammt [32]. Dennoch ist nicht auszuschließen, dass strukturelle Faktoren auch im deutschen Kontext (zumindest zukünftig) eine Rolle bei Entscheidungen für assistierten Suizid spielen könnten.

## Implikationen für die Beurteilung der Freiwilligkeit

Freiwilligkeit ist neben der Kenntnis der relevanten Informationen sowie der Einsichts- und Urteilsfähigkeit ein wesentliches Teilelement der Freiverantwortlichkeit von Entscheidungen über assistierten Suizid. Die Beurteilung der Freiwilligkeit stellt damit eine wichtige Aufgabe im Umgang mit Anfragen nach assistiertem Suizid dar. Die vorangegangene Analyse zeigt, dass Entscheidungen über assistierten Suizid vielfältigen Einflussfaktoren unterliegen können. Die dargestellten Formen interpersonaler und struktureller Einflussnahme sollen eine Orientierung darüber geben, welche Faktoren in diesem Kontext die Freiwilligkeit einschränken könnten und im konkreten Fall berücksichtigt werden sollten.

Für Mitarbeitende im Gesundheitswesen und andere Personen, die Beratungen im Zusammenhang mit assistiertem Suizid durchführen, sowie für Angehörige ist es zunächst wichtig, sich bewusst zu machen, dass sie durch eigene Aussagen möglicherweise Druck ausüben können. Eigene Hinweise, Empfehlungen und Argumente können – auch unbeabsichtigt – Einfluss auf Entscheidungen über einen assistierten Suizid nehmen. Die erläuterten Formen der Einflussnahme sollten daher, soweit möglich, nicht eingesetzt werden. Um Einschränkungen der Freiwilligkeit bestmöglich zu vermeiden und zu erkennen, erscheint zudem eine Trennung von Information und Beratung, Beurteilung der Freiverantwortlichkeit sowie der eigentlichen Suizidassistenz sinnvoll [41, 42].

Bei der Beurteilung der Freiverantwortlichkeit eines Suizidentschlusses sollte systematisch geprüft werden, ob Hinweise auf interpersonale Einflussnahmen wie Drohungen, Angebote, Überredung oder zwischenmenschliche Beeinflussung vorliegen. Hierbei kann es hilfreich sein, genau nachzufragen, welche Rolle andere Personen bei der Entscheidung spielen. Angehörige nehmen oft eine wichtige und unterstützende Funktion ein, weshalb es häufig sinnvoll ist, sie in Entscheidungsprozesse über assistierten Suizid einzubeziehen. Dennoch ist es für die Beurteilung der Freiwilligkeit auch wichtig, Gespräche ohne Angehörige zu führen, um mögliche Formen der Einflussnahme durch Angehörige selbst erkennen zu können. Wenn Hinweise auf Druck durch andere bestehen, sollten individuelle Entlastungsmöglichkeiten gesucht werden [42].

Weiterhin sollte erfragt werden, welche Rolle strukturelle Faktoren – wie beispielsweise sozioökonomische Umstände – für die Anfrage nach assistiertem Suizid spielen und ob diese die Entscheidung aus der Perspektive der Person selbst maßgeblich beeinflusst haben. In diesem Zusammenhang sollte die psychosoziale Situation der Person ausführlich erfasst werden, um auszuschließen, dass der Wunsch nach assistiertem Suizid aus mangelnden Hilfsangeboten resultiert [55].

Gleichzeitig bleibt festzuhalten, dass es häufig schwierig ist, externe Einflussnahmen sicher zu erkennen. Wie die Erläuterungen in diesem Beitrag gezeigt haben, ist es zudem herausfordernd, festzulegen, wann genau eine Einflussnahme eine Entscheidung unfreiwillig macht.

Weiterhin besteht erheblicher Forschungsbedarf in diesem Bereich: So fehlen empirische Daten zu unterschiedlichen Formen der interpersonalen Einflussnahme bei assistiertem Suizid sowie zur Rolle struktureller Faktoren. Empirische Forschung mit Personen, die eine Suizidassistenz erwägen, Angehörigen und Mitarbeitenden im Gesundheitswesen in diesem Bereich ist notwendig, um nachvollziehen zu können, welche Rolle die genannten Faktoren für Entscheidungen über einen assistierten Suizid spielen. Dies ist auch für die Entwicklung von Leitlinien, die Kriterien für die Beurteilung der Freiwilligkeit von Anfragen nach Suizidassistenz festlegen, wünschenswert.

## Fazit

Der vorliegende Beitrag hat einen Überblick über Freiwilligkeit als Element der Freiverantwortlichkeit von Entscheidungen über assistierten Suizid gegeben. Es wurden relevante Akteure genannt, die im Zusammenhang mit Anfragen nach Suizidassistenz eine Rolle spielen können – darunter Mitarbeitende im Gesundheitswesen, Sterbehelfer*innen und Angehörige. Anhand medizinethischer und philosophischer Literatur sowie empirischer Erkenntnisse aus angrenzenden Praxisfeldern wurden verschiedene Formen der Einflussnahme diskutiert und dargestellt, inwiefern strukturelle Rahmenbedingungen die Freiwilligkeit einschränken könnten.

Auf dieser Grundlage wurden erste Überlegungen zur Beurteilung der Freiwilligkeit bei Anfragen nach Suizidassistenz angestellt. Einerseits ist für die relevanten Akteure wichtig, sich bewusst zu sein, dass sie – auch unabsichtlich – Einfluss auf Entscheidungen über assistierten Suizid nehmen können, um einen solchen Einfluss möglichst vermeiden zu können. Andererseits sollte bei der Beurteilung der Freiwilligkeit systematisch erfasst werden, ob Hinweise darauf bestehen, dass Formen der äußeren Einflussnahme vorliegen. Eine Trennung von Information und Beratung, Beurteilung der Freiverantwortlichkeit und Durchführung der Suizidassistenz scheint zudem hilfreich, um Einschränkungen der Freiwilligkeit zu erkennen.

Angesichts der bislang begrenzten empirischen Datenlage ist weitere Forschung erforderlich, um ein fundiertes Verständnis darüber zu gewinnen, wie sich äußere Einflüsse auf die Freiwilligkeit von Entscheidungen zum assistierten Suizid auswirken und wie diese bei der Beurteilung der Freiwilligkeit berücksichtigt werden können. Zudem besteht weiterer konzeptioneller und ethischer Forschungsbedarf, um Kriterien für die Freiwilligkeit von Anfragen nach assistiertem Suizid herauszuarbeiten.

## Footnotes

1 Ob dies einen assistierten Suizid auch aus ethischer Perspektive bereits zulässig macht, wird in der Literatur kontrovers diskutiert [4, 5].

2 Diesbezüglich verweist das BVerfG auf seine Entscheidung zur medikamentösen Zwangsbehandlung und die dortigen Ausführungen zur Einwilligung in medizinische Maßnahmen. Dort heißt es, dass die Person keinem unzulässigen Druck ausgesetzt sein dürfe, etwa „durch das Inaussichtstellen von Nachteilen im Falle der Behandlungsverweigerung“ [10].
